# Endometrial carcinoma and immune escape: prognostic relevance of HLA class I loss in NSMP subtype

**DOI:** 10.1111/his.15531

**Published:** 2025-08-12

**Authors:** Marco Grillini, Jacopo Lenzi, Claudio Ceccarelli, Dario de Biase, Thais Maloberti, Sara Coluccelli, Laura Poppi, Riccardo Ciudino, Caterina Ravaioli, Camelia Alexandra Coadǎ, Gloria Ravegnini, Anna Myriam Perrone, Pierandrea De Iaco, Daniela Rubino, Claudio Zamagni, Benedetta Donati, Alessia Ciarrocchi, Sabrina Croce, Martin Köbel, Cheng‐Han Lee, Giovanni Tallini, Antonio De Leo

**Affiliations:** ^1^ Pathology Unit IRCCS Azienda Ospedaliero‐Universitaria di Bologna Bologna Italy; ^2^ Department of Medical and Surgical Sciences (DIMEC) University of Bologna Bologna Italy; ^3^ Department of Biomedical and Neuromotor Sciences University of Bologna Bologna Italy; ^4^ Department of Pharmacy and Biotechnology (FaBit) University of Bologna Bologna Italy; ^5^ Solid Tumor Molecular Pathology Laboratory IRCCS Azienda Ospedaliero‐Universitaria di Bologna Bologna Italy; ^6^ University of Medicine and Pharmacy “Iuliu Hatieganu” Cluj‐Napoca Romania; ^7^ Division of Oncologic Gynecology IRCCS Azienda Ospedaliero‐Universitaria di Bologna Bologna Italy; ^8^ Addarii Medical Oncology IRCCS Azienda Ospedaliero‐Universitaria di Bologna Bologna Italy; ^9^ Laboratory of Translational Research Arcispedale Santa Maria Nuova, Azienda Unità Sanitaria Locale‐IRCCS di Reggio Emilia Reggio Emilia Italy; ^10^ Department of Biopathology Institut Bergonié Bordeaux France; ^11^ Department of Pathology and Laboratory Medicine University of Calgary Calgary Alberta Canada; ^12^ Department of Laboratory Medicine and Pathology University of Alberta Edmonton Alberta Canada

**Keywords:** endometrial carcinoma, histopathological parameters, HLA‐I, immune phenotype, molecular classification, risk stratification, tumour microenvironment

## Abstract

**Aims:**

This study aims to define and characterize human leukocyte antigen class I (HLA‐I) expression in a consecutive series of molecularly classified endometrial carcinomas (ECs), and to evaluate its association with clinicopathologic features, spatial cancer–immune phenotypes and patient prognosis, with a focus on the NSMP (no specific molecular profile) subtype.

**Methods and results:**

HLA‐I expression was assessed by immunohistochemistry on whole tissue sections from 208 ECs, classified into *POLE*‐mutated, MMR‐deficient (MMRd), p53‐abnormal (p53abn) and NSMP subtypes. Loss of HLA‐I was identified in 31% of cases and was associated with adverse features including high‐grade, aggressive histotypes, deep myometrial invasion, substantial lymphovascular space invasion (LVSI), extensive tumour necrosis and an ‘excluded’ immune phenotype. While HLA‐I loss showed no significant prognostic impact in *POLE*, MMRd or p53abn tumours, it significantly correlated with worse disease‐free survival in NSMP tumours (*P* < 0.001). Multivariate analysis confirmed HLA‐I loss as an independent prognostic factor in early‐stage NSMP ECs, in addition to substantial LVSI, presence of lymph node metastases and spatial cancer–immune phenotypes. Integration of HLA‐I status improved the performance of predictive models over time.

**Conclusions:**

HLA‐I loss defines a biologically aggressive subgroup within NSMP ECs and is associated with adverse clinicopathologic and immune features. Assessment of HLA‐I expression could refine risk stratification in NSMP ECs, a group traditionally lacking robust prognostic markers and may help identify patients who could benefit from intensified clinical surveillance and future immunomodulatory treatment strategies.

## Graphical abstract

HLA‐I loss identifies a biologically aggressive subgroup within NSMP endometrial carcinomas, characterized by an ‘excluded’ immune phenotype and reduced disease‐free survival. Integrating HLA‐I status into prognostic models enhances risk stratification and may guide future immunotherapeutic strategies.

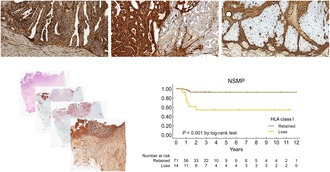

**Abbreviations**
APM: antigen presentation machinery
DFS: disease‐free survival
EC: Endometrial carcinoma
HLA: human leukocyte antigens
HPF: high‐power fields
ICIs: immune checkpoint inhibitors
iTILs: intraepithelial tumour‐lymphocytes
LOH: loss of heterozygosity
MHC: major histocompatibility complex
MELF: microcystic, elongated and fragmented
MMRd: Mismatch repair deficient
MSI-H: microsatellite instability?high
NSMP: no specific molecular profile
p53abn: p53 abnormal

## Introduction

Endometrial carcinoma (EC) is one of the most common gynaecological cancers globally, with an increasing incidence driven by ageing populations and the prevalence of metabolic disorders. Despite effective treatments for early‐stage EC, advanced or recurrent disease presents significant therapeutic challenges.^1^ Recent advances in the molecular classification of EC have significantly improved prognostic stratification and paved the way for targeted treatments such as immunotherapy. This classification identifies four distinct molecular subtypes: *POLE*‐ultramutated, microsatellite instability‐high (MSI‐H)/Mismatch repair deficient (MMRd), copy‐number low/no specific molecular profile (NSMP) and copy‐number high/p53 abnormal (p53abn).^2^ Each subtype has unique molecular features that influence prognosis and treatment response, allowing for more precise therapeutic approaches, including the use of immune checkpoint inhibitors (ICIs) for specific subtypes.

A crucial aspect of determining treatment efficacy lies in understanding tumour immune evasion. Among these mechanisms, the major histocompatibility complex (MHC) class I molecules, also known as human leukocyte antigens (HLA) in humans, have emerged as key players in the antitumour immune response.^3^, ^4^ HLA‐I molecules are essential components of the antigen presentation pathway, enabling CD8^+^ T cells to recognize and eliminate tumour cells that present neoantigens. Structurally, HLA‐I comprises an α‐heavy chain and a β2‐microglobulin light chain, which together present tumour‐specific antigens to cytotoxic T cells. This complex is assembled in the endoplasmic reticulum with the help of antigen presentation machinery (APM) components and subsequently displayed on the cell surface. Multiple factors contribute to the loss of HLA‐I expression in tumours.^5^ Genetic alterations such as loss of heterozygosity (LOH) or mutations in the β2‐microglobulin gene disrupt the structural integrity of HLA molecules.^6^ Additionally, inactivating mutations in JAK1/2 impair the IFNγ–JAK–STAT signalling cascade required for transcriptional upregulation of APM and HLA‐I. In MMRd ECs, JAK1 frameshift mutations occur in approximately 35% of cases and are associated with defective induction of both APM and HLA‐I.^7^ Epigenetic modifications, including promoter hypermethylation and histone deacetylation, further contribute to APM silencing.^3^ Finally, tumour microenvironmental factors like hypoxia and immunosuppressive cytokines (e.g. TGF‐β, IL‐10) further downregulate HLA‐I expression, while fostering a suppressive milieu dominated by regulatory T cells and M2 macrophages.^8^ Reduced or absent expression of HLA‐I on the tumour cell surface is a phenomenon observed also in EC and has significant implications for immune recognition, representing a potential resistance mechanism to immunotherapy, especially in immune‐rich tumours such as MMRd.^9^, ^10^ Previous studies report HLA‐I downregulation in half of EC cases, associated with tumour progression and poor progression‐free survival and overall survival.^11^ Through immune selection, loss of HLA‐I allows tumour cells to escape immune detection, promoting tumour growth and metastatic spread.

This study aims to define and characterize HLA‐I expression in a consecutive series of molecularly classified ECs, and to assess its correlation with clinicopathologic parameters, immune microenvironment features and patient prognosis.

## Materials and Methods

### Study Cohort and Clinicopathologic Parameters

After approval by the local ethics committee CE‐AVEC (Comitato Etico Area Vasta Emilia Centro, registration n. 27/2019/Sper/AOUBo and 10/2023/Sper/AOUBo), all 208 patients enrolled signed informed consent before surgical resection and diagnosis. Surgical hysterectomy and staging were performed at the Division of Oncologic Gynecology, ‘IRCCS Azienda Ospedaliero‐Universitaria di Bologna’ (Bologna, Italy). For each case, formalin‐fixed paraffin‐embedded (FFPE) representative blocks were obtained from the files of the Pathology Unit by an expert pathologist (A.D.L). All immunohistochemical (IHC) and molecular analyses were performed on whole tissue sections. A dedicated database was created by entering the parameters outlined hereafter: age at diagnosis, Body Mass Index (BMI), staging (International Federation of Gynaecology and Obstetrics—FIGO 2009),^12^ histological classification (WHO 2020 criteria),^13^ tumour grade,^14^ lymphovascular space invasion (LVSI) assessment (with substantial LVSI defined as ≥4 foci),^15^ myometrial invasion pattern—including microcystic, elongated and fragmented (MELF) pattern,^16^ and/or single invasive cells or small group of cells (tumour budding) classified as present or absent.^17^ Any extensive tumour necrosis (>25% of tumour) was duly recorded, excluding necrosis confined within the glands or on the surface of the tumour. Proliferation was assessed by mitotic index, defined as the number of mitotic figures per 10 high‐power fields (HPF, 400×): mitotic figures were manually counted in 10 consecutive HPFs within the most mitotically active (‘hot spot’) areas of the tumour.

### Immunohistochemistry

IHC for HLA‐I (mAb EMR8‐5, Abcam, UK), MLH1, PMS2, MSH2, MSH6, p53, ER (estrogen receptor), CD68, CD20, CD3, CD8 and PD‐L1 was performed on 3‐μm whole tissue sections (from the same FFPE blocks used for molecular analysis), using a BenchMark Ultra immunostainer (Ventana Medical Systems‐Roche Diagnostics, Switzerland). Antibodies and protocols are detailed in Table S1.

#### IHC evaluation of HLA‐I expression

**Figure 1:**
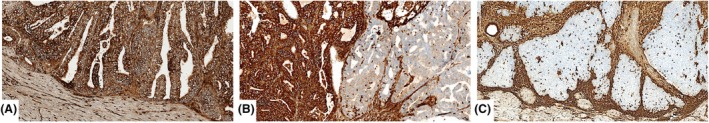
Evaluation of HLA‐I expression in endometrial carcinoma. (**A**) Intact/positive: >90% of tumour cells exhibiting membranous and/or cytoplasmic staining; (**B**) Subclonal Loss: 10%–90% of tumour cells expressing HLA‐I; (**C**) loss: <10% of tumour cells showing HLA‐I staining.

HLA‐I expression in tumours was firstly categorized as ‘intact or positive’ (>90% of tumour cells showing membranous and/or cytoplasmic staining), ‘subclonal loss’ (10%–90% of tumour cells showing expression with areas of retained HLA‐I immediately juxtaposed with areas of negative tumour staining) and ‘loss’ (<10% of tumour cells showing expression), as previously described^9^ (Figure 1). Then a simplified categorization was applied: HLA‐I ‘retained’ (formerly intact/positive expression) versus HLA‐I ‘loss’ (formerly subclonal loss/loss of expression, i.e. any loss ≥10%).

#### IHC evaluation of ER

The percentage of ER stained neoplastic cells was quantified as previously described.^18^, ^19^

#### IHC evaluation of markers for surrogate molecular classification

p53 staining was classified as normal/wild‐type or abnormal/mutant‐like if an aberrant pattern was present as follows: overexpression, absent expression or cytoplasmic staining.^20^, ^21^ Tumours were defined as MMRd according to published guidelines.^22^, ^23^

#### IHC evaluation of immune cell markers and spatial cancer‐immune phenotype (SCI) determination

SCIs were identified according to our previous publication.^24^ Immune markers evaluation was restricted to the tumour‐invasive front, segmented in its entirety at x200. Digital images were evaluated using an operator‐guided image analysis system IMAGE Pro Plus 5.1 software (Media Cybernetics Inc.). Inflamed tumours were identified by high CD8^+^ intraepithelial tumour‐infiltrating lymphocytes (iTILs) density. Excluded tumours were typified by low CD8^+^iTILs, any PD‐L1 value and a high total inflammatory component. Finally, desert tumours were characterized by low CD8^+^iTILs, PD‐L1 and low total inflammatory component values (Supplementary Material and Methods).

### Molecular Classification of Endometrial Carcinoma

Cases were classified as *POLE*, MMRd, p53abn and NSMP according to the WHO algorithm.^13^ Sequencing for *POLE* was assessed as previously described and summarized in Supplementary Material and Methods. First, only *POLE* pathogenic variants were used to assign the *POLE* subtype; then consecutive IHC analysis for MMR proteins and p53 expression was evaluated to define MMRd and p53abn tumours. Tumours with normal MMR and p53 expression and no *POLE* mutations were classified as NSMP.

### Statistical Analysis

Numerical variables were summarized as mean ± standard deviation [minimum to maximum], while categorical variables were summarized as frequencies and percentages. Crude comparisons of baseline clinicopathologic characteristics across HLA‐I expression were performed with *t*‐test, chi‐squared test or Fisher's exact test, when appropriate. All *t*‐tests were permutation‐based, with 10,000 Monte Carlo replications, to ensure robustness to moderate sample size, outliers and heteroskedasticity. Comparisons of baseline clinicopathologic characteristics were descriptive in nature and not adjusted for multiplicity, in line with recommendations for exploratory studies. Comparisons of immune cell markers by HLA‐I expression were adjusted using the Benjamini–Hochberg method to control the false discovery rate.

The Kaplan–Meier estimator was used to display the time to relapse after surgery according to HLA‐I status and/or molecular subtype; the equality of survivor functions was assessed using the log‐rank test. Loss to follow‐up and study ending were treated as right‐censored data; no unrelated premature deaths occurred. Cox proportional‐hazards regression analysis was used to investigate the association of significant baseline characteristics with disease‐free survival (DFS), selecting such characteristics from an initial pool of potential predictors with an automated stepwise procedure with significance levels of removal and addition equal to 0.05. HLA‐I expression was then added as an additional covariate, and its contribution to the model fit (i.e. prediction accuracy) was quantified by means of the time‐dependent Brier score obtained via the inverse probability of censoring weighting.^25^ All analyses were carried out using Stata (version 17, StataCorp, College Station, TX, USA). Significance was set at *P* < 0.05.

## Results

### Loss of Expression of HLA‐I in Endometrial Carcinoma and Clinicopathologic Characteristics

**Figure 2:**
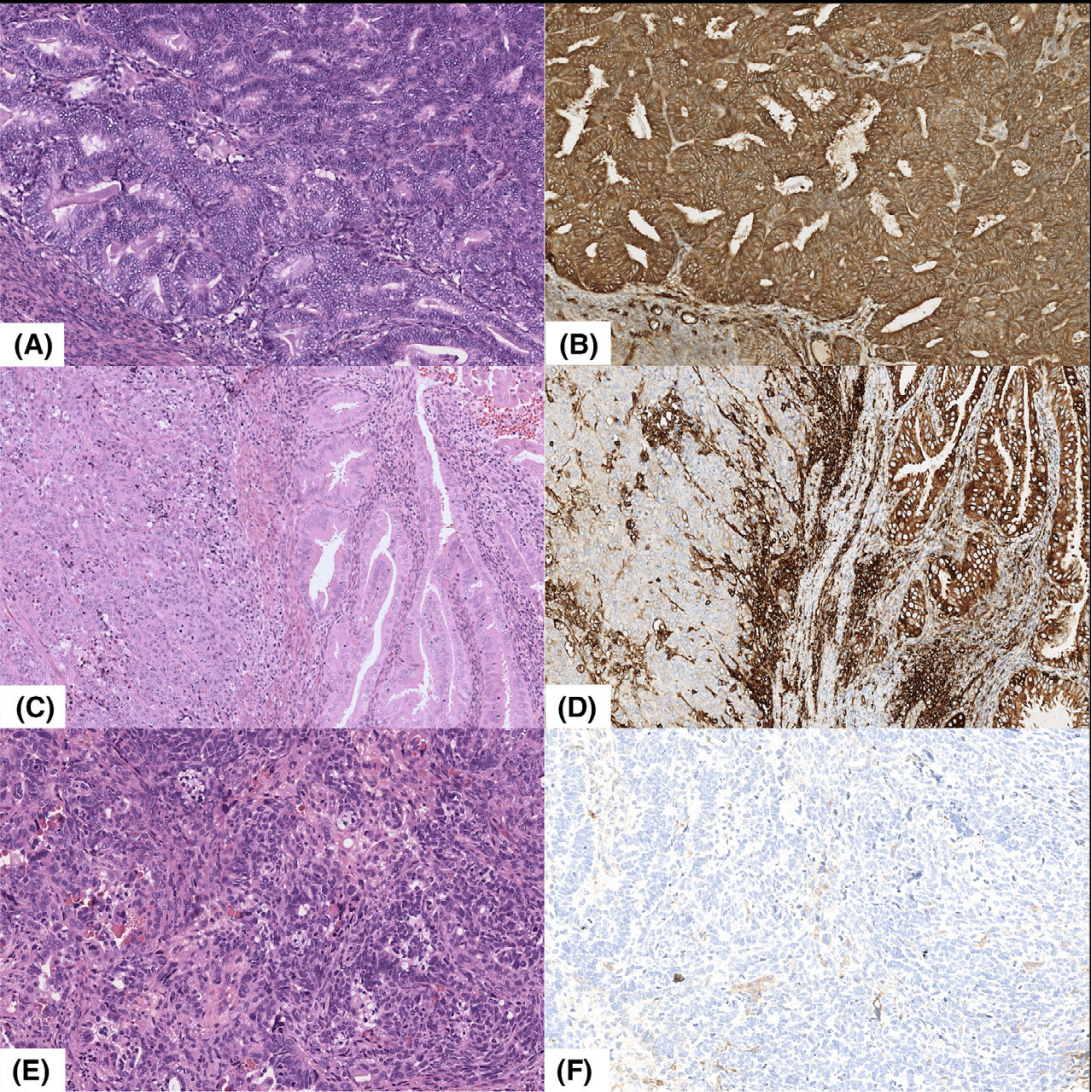
(**A** and **B**) Low‐grade endometrioid carcinoma with retained HLA‐I expression (H&E and IHC, ×200 magnification). (**C** and **D**) Dedifferentiated carcinoma with loss of HLA‐I expression: retained expression in the differentiated component and complete loss in the undifferentiated component (H&E and IHC, ×200 magnification). (**E** and **F**) Carcinosarcoma showing loss of HLA‐I expression in both the carcinomatous and sarcomatous components (H&E and IHC, ×200 magnification).

HLA‐I expression was evaluated on 208 ECs, which included 161 endometrioid carcinomas, 16 serous carcinomas, 3 clear cell carcinomas, 24 undifferentiated/dedifferentiated carcinomas and 4 carcinosarcomas (Table 1). Loss of HLA‐I expression was observed in 45 of 161 endometrioid carcinomas (25 subclonal), 4 of 16 serous carcinomas (3 subclonal), 13 of 24 undifferentiated/dedifferentiated carcinomas (10 subclonal) and 3 of 4 carcinosarcomas (none subclonal). In eight dedifferentiated carcinomas showing HLA‐I loss, the undifferentiated carcinoma component showed HLA‐I loss in all cases whereas the corresponding differentiated carcinoma component showed HLA‐I loss in three of eight cases. In carcinosarcomas showing HLA‐I loss, HLA‐I loss was observed in both the carcinomatous and the sarcomatous components (Figure 2).

**Table 1:** Clinicopathologic characteristics of the study sample, overall and by HLA‐I expression

Characteristics	All (*n* = 208)	HLA‐I expression		
		Retained	Loss	*P*‐value
		(*n* = 143)	(*n* = 65)	
Age, years	62.6 ± 10.5	62.7 ± 10.7	62.2 ± 10.1	0.73
	[34–86]	[36–86]	[34–78]	
BMI, kg/m^2^	28.1 ± 7.3	28.5 ± 7.8	27.4 ± 5.9	0.32
	[18.3–55.3]	[18.3–55.3]	[19.0–55.0]	
Histotype				
Endometrioid carcinoma	161 (77.4%)	116 (81.1%)	45 (69.2%)	0.02*
Undifferentiated/dedifferentiated carcinoma	24 (11.5%)	11 (7.7%)	13 (20.0%)	
Serous carcinoma	16 (7.7%)	12 (8.4%)	4 (6.2%)	
Carcinosarcoma	4 (1.9%)	1 (0.7%)	3 (4.6%)	
Clear cell carcinoma	3 (1.4%)	3 (2.1%)	0 (0.0%)	
Molecular subtype				
*POLE*	16 (7.7%)	4 (2.8%)	12 (18.5%)	<0.001*
MMRd	66 (31.7%)	37 (25.9%)	29 (44.6%)	
p53abn	41 (19.7%)	31 (21.7%)	10 (15.4%)	
NSMP	85 (40.9%)	71 (49.7%)	14 (21.5%)	
Grade				
Low	123 (59.1%)	93 (65.0%)	30 (46.2%)	0.01*
High	85 (40.9%)	50 (35.0%)	35 (53.8%)	
ER status, %	72.8 ± 34.1	75.5 ± 32.2	66.8 ± 37.6	0.09
	[0–100]	[0–100]	[0–100]	
Depth of invasion				
<50%	148 (71.2%)	111 (77.6%)	37 (56.9%)	0.002*
≥50%	60 (28.8%)	32 (22.4%)	28 (43.1%)	
Lymph node status				
Negative	173 (83.2%)	124 (86.7%)	49 (75.4%)	0.06
Positive	31 (14.9%)	16 (11.2%)	15 (23.1%)	
Unknown	4 (1.9%)	3 (2.1%)	1 (1.5%)	
FIGO stage 2009				
IA	120 (57.7%)	94 (65.7%)	26 (40.0%)	0.002*
IB/II	41 (19.7%)	23 (16.1%)	18 (27.7%)	
III	38 (18.3%)	19 (13.3%)	19 (29.2%)	
IV	9 (4.3%)	7 (4.9%)	2 (3.1%)	
Mitoses, 10 HPFs	52.6 ± 36.4	46.5 ± 35.7	65.8 ± 34.6	<0.001*
	[1–230]	[1–230]	[2–150]	
Extensive tumour necrosis				
Absent	104 (50.0%)	90 (62.9%)	14 (21.5%)	<0.001*
Present	104 (50.0%)	53 (37.1%)	51 (78.5%)	
MELF pattern of invasion				
Negative	139 (66.8%)	96 (67.1%)	43 (66.2%)	0.89
Positive	69 (33.2%)	47 (32.9%)	22 (33.8%)	
Tumour budding				
Absent	119 (57.2%)	88 (61.5%)	31 (47.7%)	0.06
Present	89 (42.8%)	55 (38.5%)	34 (52.3%)	
LVSI				
Absent/focal	133 (63.9%)	102 (71.3%)	31 (47.7%)	0.001*
Substantial	75 (36.1%)	41 (28.7%)	34 (52.3%)	
Spatial cancer‐immune phenotype				
Inflamed	103 (49.5%)	73 (51.0%)	30 (46.2%)	0.03*
Desert	60 (28.9%)	46 (32.2%)	14 (21.5%)	
Excluded	45 (21.6%)	24 (16.8%)	21 (32.3%)	

Values are *n* (%) or mean ± standard deviation [minimum to maximum].

ER, estrogen receptor; FIGO, 2009 International Federation of Gynaecology and Obstetrics; HPF, high‐power field; LVSI, lymphovascular space invasion; MELF, microcystic, elongated, fragmented; MMRd, mismatch repair deficient; NSMP, no specific molecular profile; p53abn, *TP53* mutant; *POLE*, *POLE* mutant.

* *p*‐value ≤ 0.05.

By molecular subtype, HLA‐I loss was observed in 75% of *POLE* and 44% of MMRd ECs, while being less common in p53abn (24%) and NSMP tumours (16%) (Table 1).

Median follow‐up was 35.0 months (1–144 months) and 35/208 (16.8%) patients recurred.

The overall clinicopathologic characteristics, molecular subtypes and HLA‐I expression of the 208 EC patients are summarized in Table 1. HLA‐I loss of expression was mainly associated with aggressive histotypes (undifferentiated/dedifferentiated and carcinosarcoma), high‐grade tumours, deeper myometrial invasion, advanced stage, extensive tumour necrosis, higher mitotic count and substantial LVSI. There was also a trend towards association with lymph node metastasis and tumour budding, although not statistically significant. Considering SCI classification, HLA‐I loss was most marked in the excluded phenotype.

### HLA‐I and Immune Cell Markers

**Figure 3:**
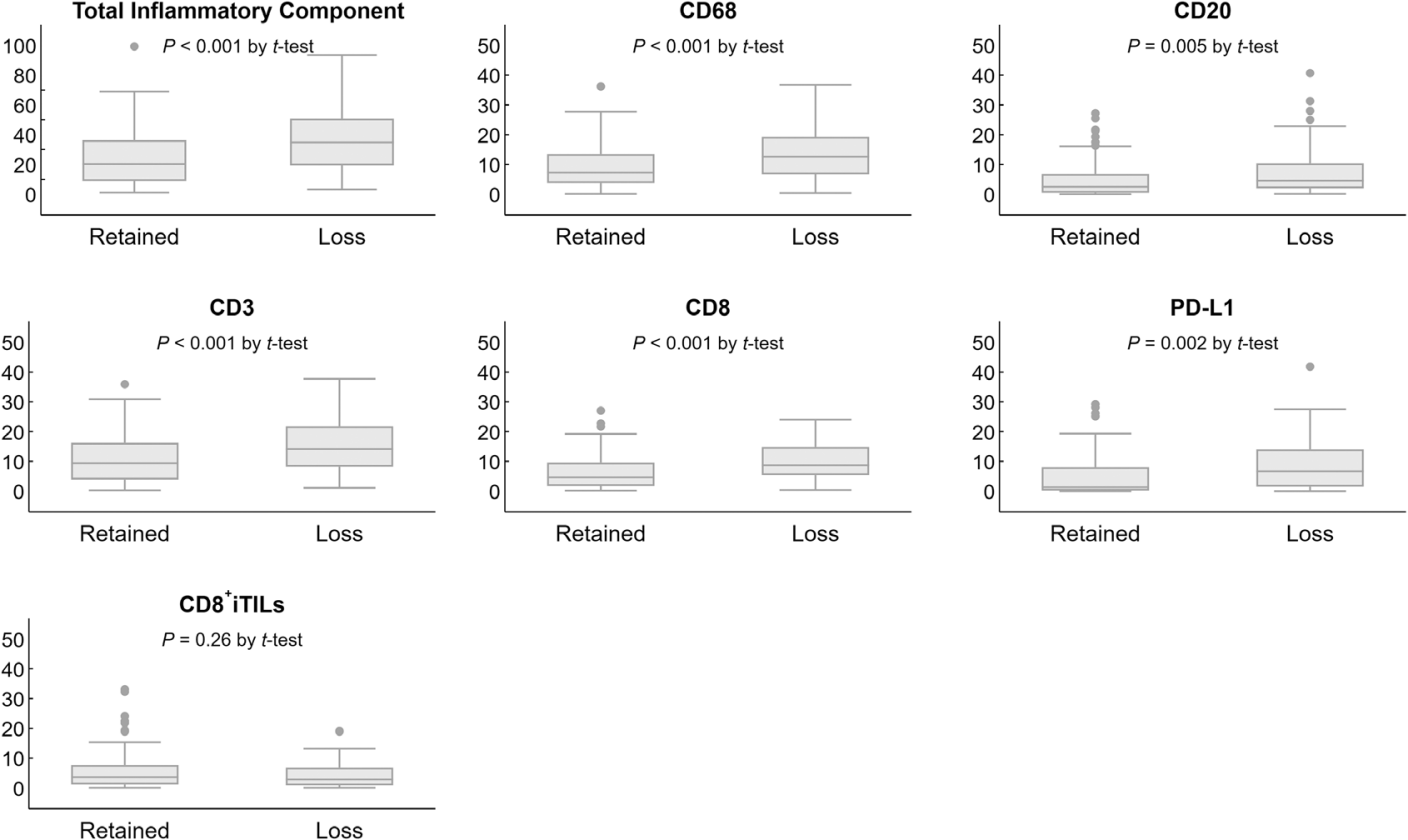
Box‐and‐whisker plots showing the distribution of immune cell markers by HLA‐I expression.

Loss of HLA‐I was associated with an increased peri‐tumoral inflammatory component, either considered as the total inflammatory component or subdivided into individual immune cell markers (CD20, CD3 and CD68). Higher PD‐L1 expression in dendritic and macrophage components was also observed in tumours with HLA‐I loss, consistent with features of the excluded immune phenotype. In contrast, CD8^+^iTILs did not differ based on HLA‐I status (Figure 3).

### Prognostic Impact of HLA‐I Expression

Although HLA‐I expression was not significantly associated with recurrence overall, a non‐significant trend towards worse prognosis was observed in cases with HLA‐I loss (Figure S1).

**Figure 4:**
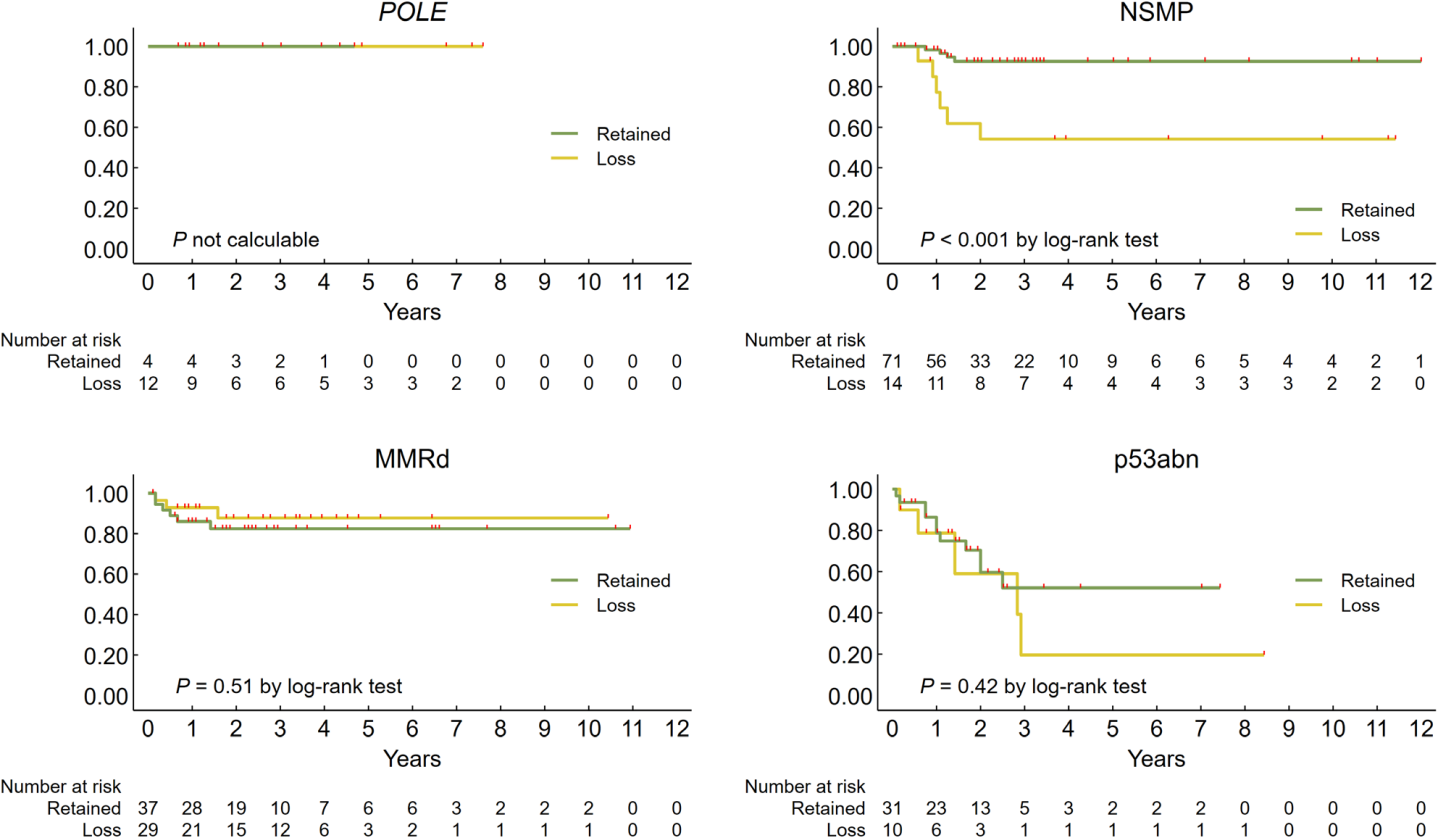
Kaplan–Meier estimates of disease‐free survival according to HLA‐I expression stratified by molecular subtype; censoring times are marked with red spikes.

In stratified analysis by molecular subtype, loss of HLA‐I expression was not significantly associated with DFS in *POLE*, MMRd and p53abn subtypes. However, in the NSMP subtype, loss of HLA‐I was strongly associated with tumour recurrence (log‐rank *P* < 0.001) (Figure 4).

**Figure 5:**
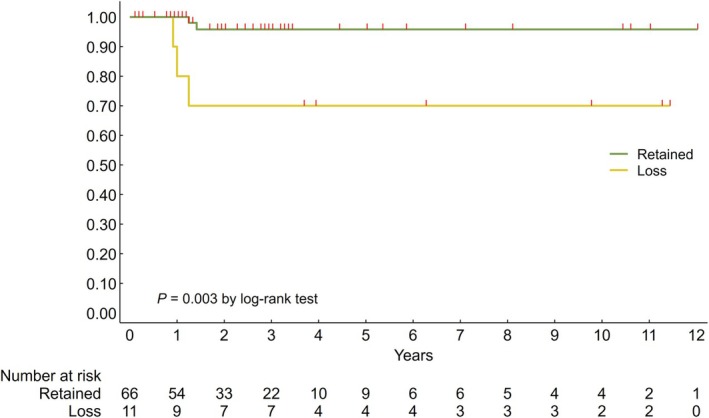
Kaplan–Meier estimates of disease‐free survival according to HLA‐I expression in NSMP tumours with early stages; censoring times are marked with red spikes.

In the overall cohort, considering FIGO 2009 stages I and II ECs (*n* = 161), HLA‐I loss correlated with a worse prognosis (*P* = 0.04) (Figure S2). When the analysis was further restricted to NSMP tumours with FIGO 2009 stages I and II (*n* = 77), the significant difference between HLA‐I loss versus retained (*P* = 0.003) was confirmed also in this setting (early staged NSMP ECs) (Figure 5).

Multivariate analysis to estimate the prognostic value of loss of HLA‐I expression for the entire cohort in the context of other selected covariates (i.e. histotype, grade, FIGO 2009 stage, LVSI and molecular subtype) shows that FIGO 2009 stage, tumour grade and tumour budding were independent predictive parameters of DFS, while HLA‐I and SCI were not statistically significant (Table 2).

**Table 2:** Cox proportional hazards regression analysis of disease‐free survival in the overall sample of 208 patients. The outputs are presented progressively, including spatial cancer‐immune phenotype and HLA‐I expression in the model. Mean Brier scores at 12, 24, 36 and 48 months are provided at the bottom of the table

Characteristics	Without spatial cancer‐immune phenotype and HLA‐I			With spatial cancer‐immune phenotype			With HLA‐I			With spatial cancer‐immune phenotype and HLA‐I		
	Hazard ratio	*P*‐value	95% CI	Hazard ratio	*P*‐value	95% CI	Hazard ratio	*P*‐value	95% CI	Hazard ratio	*P*‐value	95% CI
FIGO stage 2009												
IA	Ref.			Ref.			Ref.			Ref.		
IB/II	1.44	0.52	0.47–4.41	1.53	0.46	0.50–4.71	1.52	0.47	0.49–4.70	1.62	0.040	0.52–5.05
III	6.60	<0.001	2.65–16.44	6.72	<0.001	2.66–16.94	7.26	<0.001	2.73–19.32	7.49	<0.001	2.77–20.23
IV	15.71	<0.001	4.32–57.08	14.73	<0.001	4.02–53.98	15.73	<0.001	4.30–57.55	14.75	<0.001	4.01–54.29
Tumour grade												
Low	Ref.			Ref.			Ref.			Ref.		
High	2.86	0.01	1.25–6.52	2.64	0.03	1.13–6.15	2.86	0.01	1.25–6.54	2.61	0.03	1.11–6.13
Tumour budding												
Absent	Ref.			Ref.			Ref.			Ref.		
Present	0.47	0.04	0.23–0.96	0.50	0.05	0.26–1.00	0.47	0.04	0.23–0.96	0.49	0.05	0.26–1.00
Spatial cancer‐immune phenotype												
Inflamed	Ref.			Ref.			Ref.			Ref.		
Desert	··	··	··	1.54	0.32	0.65–3.61	··	··	··	1.55	0.31	0.66–3.64
Excluded	··	··	··	1.28	0.57	0.55–2.96	··	··	··	1.32	0.52	0.56–3.08
HLA‐I expression												
Retained	Ref.			Ref.			Ref.			Ref.		
Loss	··	··	··	··	··	··	0.80	0.57	0.38–1.71	0.78	0.53	0.37–1.68
Brier score												
12 months	0.070		0.043–0.097	0.072		0.044–0.100	0.069		0.042–0.096	0.071		0.043–0.099
24 months	0.110		0.074–0.146	0.111		0.075–0.147	0.107		0.072–0.142	0.109		0.073–0.144
36 months	0.125		0.082–0.167	0.131		0.082–0.181	0.119		0.079–0.160	0.124		0.079–0.168
48 months	0.127		0.082–0.173	0.136		0.082–0.190	0.122		0.079–0.164	0.128		0.080–0.176

FIGO stage, tumour grade and budding were selected as significant predictors of disease‐free survival using an automated stepwise procedure with significance levels of removal and addition equal to 0.05. Mid‐dots (··) mean that the variable was voluntarily discarded from the model.

CI, confidence interval; FIGO, International Federation of Gynaecology and Obstetrics.

In multivariate analysis restricted to the NSMP molecular subtype (Table 3), conventional pathological features significantly associated with DFS included ER status, lymph node status and substantial LVSI. However, when HLA‐I expression and SCIs were incorporated into the model, these immune‐related variables emerged as independent prognostic factors. In this adjusted model, ER status lost statistical significance, lymph node involvement became only borderline significant, while substantial LVSI remained a strong and independent predictor of recurrence. Notably, loss of HLA‐I expression (HR = 24.63, *P* = 0.007, 95% CI = 2.40–257.27) identified a subgroup of tumours with a markedly increased risk of recurrence, independently of the type of SCI.

**Table 3:** Cox proportional hazards regression analysis of disease‐free survival in the NSMP molecular subtype. The outputs are presented progressively, including spatial cancer‐immune phenotype and HLA‐I expression in the model. Mean Brier scores at 12, 24, 36 and 48 months are provided at the bottom of the table

Characteristics	Without spatial cancer‐immune phenotype and HLA‐I			With spatial cancer‐immune phenotype			With HLA‐I			With spatial cancer‐immune phenotype and HLA‐I		
	Hazard ratio	*P*‐value	95% CI	Hazard ratio	*P*‐value	95% CI	Hazard ratio	*P*‐value	95% CI	Hazard ratio	*P*‐value	95% CI
ER expression, %	0.97	0.004	0.95–0.99	0.97	0.005	0.94–0.99	0.97	0.002	0.96–0.99	Ref.		
Lymph node status										··	··	··
Negative	Ref.			Ref.			Ref.			Ref.		
Positive	4.69	0.04	1.05–20.82	14.67	0.009	1.98–108.68	··	··	··	6.63	0.05	1.01–43.49
Extensive tumour necrosis												
Absent	Ref.			Ref.			Ref.			Ref.		
Present	12.80	0.02	1.43–114.37	··	··	··	··	··	··	··	··	··
LVSI												
Absent/Focal	Ref.			Ref.			Ref.			Ref.		
Substantial	6.53	0.02	1.44–29.60	16.26	0.001	3.30–80.15	8.98	0.002	2.18–37.01	80.95	<0.001	6.89–950.80
Spatial cancer‐immune phenotype												
Inflamed	Ref.			Ref.			Ref.			Ref.		
Desert	··	··	··	42.76	0.008	2.62–697.62	··	··	··	380.43	0.001	10.70–>10,000
Excluded	··	··	··	53.50	0.005	3.35–854.96	··	··	··	280.16	0.003	6.65–>10,000
HLA‐I expression												
Retained	Ref.			Ref.			Ref.			Ref.		
Loss	··	··	··	··	··	··	8.68	0.002	2.18–34.63	24.63	0.007	2.40–252.27
Brier score												
12 months	0.023		0.000–0.050	0.026		0.000–0.057	0.023		0.000–0.051	0.032		0.000–0.069
24 months	0.044		0.003–0.084	0.039		0.002–0.076	0.080		0.022–0.138	0.024		0.007–0.040
36 months	0.047		0.004–0.090	0.041		0.003–0.079	0.105		0.011–0.200	0.026		0.008–0.044
48 months	0.042		0.001–0.082	0.036		0.000–0.073	0.065		0.015–0.114	0.024		0.005–0.043

One patient with unknown lymph node involvement was excluded. Percentage of ER, lymph node status, extensive tumour necrosis and LVSI were selected as significant predictors of disease‐free survival using an automated stepwise procedure with significance levels of removal and addition equal to 0.05. Mid‐dots (··) mean that the variable was voluntarily discarded from the model (extensive necrosis failed to achieve statistical significance when either immune phenotype or HLA‐I expression were included in the model, and was thus removed; lymph node status was not significant after the inclusion of HLA‐I expression; ER status was no longer statistically significant when both immune phenotype and HLA‐I expression were included in the model).

CI, confidence interval; ER, estrogen receptor; LVSI, lymphovascular space invasion.

**Figure 6:**
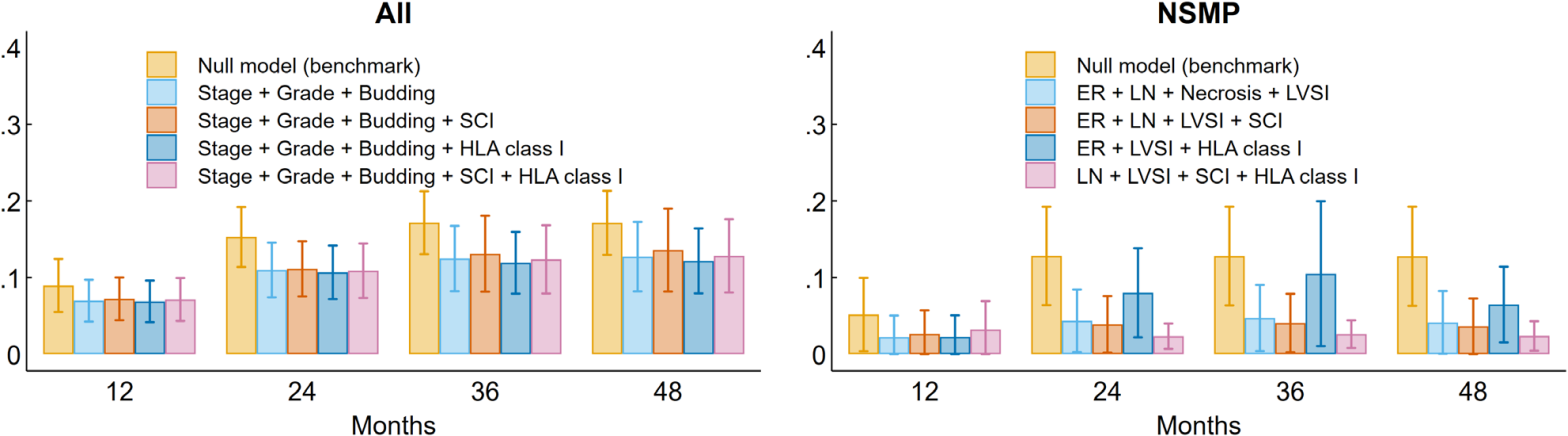
Brier scores of semi‐parametric Cox regression models investigating disease‐free survival and including different sets of predictors, starting from an empty (null) model that serves as a benchmark, overall and in the NSMP subtype. Mean scores with 95% confidence intervals are computed at 12, 24, 36 and 48 months of follow‐up. FIGO 2009 stage, tumour grade, tumour budding, ER status, LN involvement, extensive tumour necrosis and LVSI were selected as significant predictors of disease‐free survival using an automated stepwise procedure with significance levels of removal and addition equal to 0.05. In the NSMP‐specific models, extensive tumour necrosis, LN involvement and ER expression were removed after adjusting for immune phenotype and/or HLA‐I expression. ER, estrogen receptor; FIGO, International Federation of Gynaecology and Obstetrics; LN, lymph node involvement; LVSI, lymphovascular space invasion; SCI, spatial‐cancer immune phenotype.

Brier scores of semiparametric Cox regression models analysing DFS and different sets of predictors confirmed that HLA‐I expression improves the prediction model after 2 years of follow‐up in the NSMP subtype, maintaining this prognostic impact over time (36 and 48 months) (Figure 6).

## Discussion

Our study shows for the first time that loss of HLA‐I expression is associated with shorter disease‐specific survival in NSMP EC. Downregulation of HLA‐I represents an important mechanism of tumour immune evasion, allowing tumour cells to avoid CD8^+^ T cell‐mediated cytotoxicity by impairing tumour neoantigen presentation.^26^

In our study, HLA‐I loss occurred in 31% (65/208) of ECs, a prevalence comparable to the 42% reported in a previous series of 76 ECs.^9^ HLA‐I loss was more frequently observed, and it was not only enriched in tumours with aggressive histotype, such as dedifferentiated carcinomas and carcinosarcomas, but also in the MMRd molecular subtypes, confirming earlier findings.^9^ The mechanisms of HLA‐I loss are diverse, including copy number loss, mutations affecting the complex, transcriptional downregulation and post‐translational dysregulation.^27^, ^28^, ^29^, ^30^, ^31^ Although beyond the scope of the current manuscript, the high prevalence in seemingly unrelated carcinosarcomas and MMRd tumours, as well as the high prevalence of partial loss, may allow us to speculate on different mechanisms in different instances. For example, a higher likelihood of transcriptional regulation in partial loss versus more generic hard‐wired alterations in carcinosarcomas via copy number changes of the HLA‐I gene, or point mutations in MMRd ECs in genes such as β2‐microglobulin^28^ affecting the complex. This may have important implications for therapeutic approaches because HLA‐I expression may be restored in some tumours.^32^

Despite its high prevalence in MMRd ECs, HLA‐I loss was not associated with survival in these tumours, suggesting that their high immune activation due to neoantigen generation in the context of a hypermutated genotype is not altered, in survival‐relevant terms, by HLA‐I loss. In contrast, HLA‐I loss was a much higher risk of recurrence in NSMP ECs, also when stratified only for stage I/II cases. This points to a particularly important role of HLA‐I in NSMP ECs.

While the overall immune response—assessed by the total inflammatory component and its individual cell types—was significantly higher in ECs with HLA‐I loss, our analysis showed an association between HLA‐I loss and the excluded immune phenotype, which is characterized by an abundant peritumoral inflammatory component rich in CD68^+^/PD‐L1^+^ macrophages. Such ‘non‐permissive’ immune architecture mirrors what has been described in other solid tumours with HLA‐I loss and supports macrophage‐mediated immune suppression as a companion escape route.^33^ In contrast to Friedman *et al*. who reported reduced intratumoral CD8^+^ T cells in HLA‐I‐negative tumours,^9^ our spatial analyses did not reveal a significant difference in CD8^+^iTILs between HLA‐I loss and HLA‐I retained ECs. While one may expect that HLA‐I loss would reduce CD8^+^ T cell infiltration, the relationship is more complex. A compensatory response of NK cells, the innate immune system (e.g. macrophages) or microenvironment (e.g. IFNγ) may lead to an overall increase in immune cells with T cells being dysfunctional.^34^ Our spatially resolved analysis provides additional support for this theory: a predominance of the excluded immune phenotype in HLA‐loss tumours, indicating a dysfunctional immune microenvironment. Furthermore, HLA‐I loss may be an explanation for our previously described excluded immune phenotype in EC.^24^

As known, NSMP tumours represent the most common molecular subtype of EC, yet they are notably heterogeneous in clinical behaviour and lack robust prognostic stratification tools. Although recent efforts, such as subclassification based on ER and tumour grade, have improved risk prediction, these features alone may be insufficient to capture the biological complexity of NSMP tumours behaviour.^35^, ^36^ In our multivariate model, ER status—initially associated with DFS—lost statistical significance upon inclusion of immune parameters such as HLA‐I expression and SCIs, suggesting that these immune‐related features may provide additional and potentially more informative prognostic insights within the NSMP subtype. Notably, HLA‐I loss and substantial LVSI remained strong, independent predictors of recurrence, while lymph node involvement was only borderline significant. This supports the hypothesis that immune escape mechanisms and immune spatial contexture provide additional, clinically relevant stratification beyond conventional pathological parameters. Our results support the use of a composite panel of immune‐related markers that better reflects the tumour–microenvironment interplay, offering a more precise and clinically useful stratification. This approach could ultimately improve patient management even in early‐stage NSMP tumours.

Our findings also complement recent data from Grau Bejar *et al*.^37^ who investigated immune predictors of response to ICIs in MMRd ECs. Their study demonstrated that loss of HLA‐I, particularly due to β2‐microglobulin mutations or LOH, correlated with resistance to ICIs, despite the presence of a typically inflamed immune microenvironment. While their focus was on the predictive role of HLA‐I loss in MMRd tumours undergoing immunotherapy, our study extends this perspective to the prognostic impact of HLA‐I loss in NSMP ECs, a subgroup where reliable biomarkers are particularly needed.

One of the major strengths of this study lies in the use of whole‐slide, serial IHC sections rather than tissue microarrays, enabling a more accurate and spatially resolved assessment of HLA‐I expression and immune marker distribution, minimizing sampling bias.^24^ However, several limitations should be acknowledged. The retrospective, single‐centre design may limit the broader applicability of our findings, and the relatively short median follow‐up (35 months) may underestimate long‐term recurrence risk. In addition, the use of DFS as an endpoint may not fully reflect the clinical relevance of recurrence patterns in endometrial carcinoma, as local and distant relapses have different prognostic implications. Moreover, the underlying genetic or epigenetic mechanisms of HLA‐I loss were not explored, and functional correlates of immune escape were beyond the scope of this study. Future studies should aim to validate these findings in larger, multi‐institutional cohorts with longer follow‐up and to further investigate the molecular mechanisms underlying HLA‐I loss and its contribution to immune escape.

## Conclusion

Our study identifies HLA‐I loss as a biologically relevant and prognostically significant feature in NSMP ECs. Routine assessment of HLA‐I expression could improve risk stratification in this heterogeneous subtype and help identify patients who may benefit from intensified surveillance and future tailored therapeutic approaches.

## Author contributions

A.D.L., M.G., J.L. and C.C. provided study concept and design; C.C., J.L., D.d.B., T.M., S.C., R.C., C.R., M.G., C.A.C., G.R., A.M.P., P.D.I., D.R., C.Z., B.D. and A.C. provided acquisition of data, analysis and interpretation of data; A.D.L., D.d.B. and C.C. drafted the manuscript; M.K., C.H.L., S.Cr. and G.T. were involved in critical revision of the manuscript for important intellectual content; A.D.L. obtained funding; M.G., D.d.B., J.L., T.M. and C.A.C. provided technical or material support; and G.T. was involved in study supervision. All authors read and approved the final paper.

## Funding information

The research leading to these results has received funding from AIRC under MFAG 2021—ID 26319 project—P.I. De Leo Antonio.

## Conflict of interest

The authors report no relevant conflicts of interest.

## Supporting information


**Figure S1.** Kaplan–Meier estimates of disease‐free survival according to HLA class I expression in the entire cohort; censoring times are marked with red spikes.
**Figure S2.** Kaplan–Meier estimates of disease‐free survival restricted to FIGO 2009 stages I and II (*n* = 161) according to HLA class I expression; censoring times are marked with red spikes.
**Table S1.** List of antibodies and protocols.
Supplementary Material and Methods.

## Data Availability

The data that support the findings of this study are available from the corresponding author upon reasonable request.
