# Supplementary material for: Endometrial carcinoma and immune escape: prognostic relevance of HLA class I loss in NSMP subtype
Source: Histopathology. 2025 Aug 12;88(2):442–56. doi: 10.1111/his.15531 (PMC12703446; doi:10.1111/his.15531)

| **Test** | Specie | clone | **Antigen retrieval** | **Incubation** | **Visualization** |
| --- | --- | --- | --- | --- | --- |
| HLA-I | mouse | EMR8-5 | UltraCC195°Cx 24' | 36°C x 20’ | OptiView DAB |
| CD20  CD3 | Mouse  Rabbit | L26  2GV6 | Ultra CC1 95°C x 32' | 36°C x 8' (CD20)  36°C x 16' (CD3) | OptiView DAB  UltraView Red |
| CD8 | Rabbit | SP57 | Ultra CC1 95°C x 24' | 36°C x 16' | OptiView DAB |
| ER | Rabbit | SP1 | Ultra CC1 95°C x 32' | 36°C x 24' | OptiView DAB |
| MLH1 | Mouse | M1 | Ultra CC1 98°C x 56' | 36°C x 32' | OptiView DAB + Amp |
| MSH2 | Mouse | G219-1129 | Ultra CC1 95°C x 56' | 36°C x 32' | OptiView DAB |
| MSH6 | Rabbit | SP93 | Ultra CC1 100°C x 64' | 36°C x 12' | OptiView DAB |
| PMS2 | Mouse | A16-4 | Ultra CC1 99°C x 64' | 36°C x 32' | OptiView DAB + Amp |
| p53 | Mouse | DO-7 | Ultra CC1 95°C x 24' | 36°C x 12' | OptiView DAB |
| PD-L1  CD68 | Rabbit  Mouse | SP263  PG-M1 | Ultra CC1 99°C x 64' | 36°C x 12' (PD-L1)  36°C x 24' (CD68) | OptiView DAB  UltraView Red |

**Supplementary Table 1. List of antibodies and protocols**

*Notes*: All antibodies were from Ventana Medical Systems other than CD68 (Diagnostic Biosystems USA) and HLA-I (Abcam, UK)

RT = Room temperature; OptiView DAB = OptiView DAB Detection kit; UltraView RED = UltraView AP Red Detection kit; Amp = OptiView Amplification kit

PD-L1/CD68 and CD20/CD3 double staining was conducted using OptiView DAB detection kit first (brown color), then UltraView RED AP detection kit (red color). The single staining tests were performed with DAB kit alone. All sections were then counterstained using Hematoxylin and bluing reagent according to Ventana protocols.

**Supplementary Material and Methods**

***Spatial Cancer-Immunephenotype determination***

For PD-L1, CD68, CD20, and CD3, the percentage of immunostained cells was evaluated on the entire tumor invasive front on representative whole slide of the tumor on a Zeiss Axiskop 40 microscope (ocular 10/23 – objective Acroplan20x/0.45) (Carl Zeiss AG Oberkochen, Germany) using the tumor invasive front as the top side for a depth equal to each entire 200x field toward the peritumoral stroma. For each sample, the mean percentage of immunopositive cells by marker was obtained as follows: mean immunopositive area (pixels)/total field area (pixels). The percentage of the whole assessed immune population (total inflammatory component) was obtained by adding CD68, CD20, and CD3 values. Intraepithelial Tumor-Infiltrating CD8^+^ Lymphocytes (CD8^+^iTILs) index was obtained by examining the entire tumoral area at 10x magnification, selecting five hotspot fields with a higher number of intratumoralCD8-positive lymphocytes. In these fields, the percentage of iTILs was calculated as the ratio of CD8-positive intratumoral lymphocytes to the tumor cells at 200x. For each of the aforementioned markers, a classification into two classes (low and high) was obtained using the respective median as cut-off value (total inflammatory component: 23.9%; PD-L1: 2.2%; CD8^+^iTILs: 3.55%). The set of classes was then combined to obtain a categorization into the three SCIs: immune-desert, -excluded, -inflamed as follows: Desert = low/absent total inflammatory component, low PD-L1, low CD8^+^iTILs; Excluded = high total inflammatory component, low/high PD-L1, low CD8^+^iTILs; Inflamed = low/high total inflammatory component, low/high PD-L1, high CD8^+^iTILs. The application of these criteria avoided ambiguity in SCI classification. In addition, for the simplified approach the following median cut-off value were also used (CD68: 9.3%; CD20: 3.3%) and combined as follows: Desert = low CD68/CD20/PD-L1/CD8^+^iTILs; Excluded = high CD68 and/or high CD20 and/or high PD-L1, low CD8^+^iTILs; Inflamed = high CD8^+^iTILs.

***DNA extraction and Next-Generation Sequencing***

DNA was extracted from formalin-fixed paraffin-embedded tissue starting from 2 to 4 10-μm thick sections, according to the amount of tissue present in the paraffin block. The areas of interest were marked on the control hematoxylin and eosin-stained slide and manually dissected under microscopic guidance using a sterile blade. DNA was extracted using the Quick Extract Kit (LGCBiosearch Technologies) and quantified using the “Qubit” fluorometer (ThermoFisher Scientific). Samples were analyzed using a laboratory-developed multigene next-generation sequencing (NGS) panel of genomic regions and sequenced using the Gene Studio S5 Prime sequencer (ThermoFisher Scientific), according to the manufacturer’s instruction as previously published ^38^. Template preparation was performed using the Chef Machine instrument (ThermoFisher Scientific) and then sequenced using an Ion 530 chip. The panel included a total of 169 amplicons within the following gene regions (human reference sequence hg19/GRCh37, 12.74kb): ARID1A (complete coding sequence – CDS), BRAF (exon 15), KIT (exons 8, 9, 11, 13, 17), CTNNB1 (exons 3, 7, 8), HRAS (exons 2-4), KRAS (exons 2-4), NRAS (exons 2-4), PIK3CA (exons 10, 21), POLE (exons 9-14), TERT (promoter region), and TP53 (exons 2-9). Only nucleotide variations observed in at least 5% of the total number of reads analyzed were considered for mutational call. The sequences obtained were analyzed using the Ion Reporter Software (version 5.18, ThermoFisher Scientific) and the Integrative Genomics Viewer 2.12.2 (IGV) tool (Available online: http://software.broadinstitute.org/software/igv/). The Varsome tool (https://varsome.com/)^39^ was used to evaluate the classification of each mutation. POLE variants were evaluated according to Leon-Castillo et al paper indications ^40^.

***Statistical Analysis***

The Brier score is an evaluation metric of disagreement computed as the average squared distance between the observed survival status and the predicted survival probability at specific time points, and is always a number between 0 and 1, with 0 being the best possible value that indicates perfect fit. The proportional-hazards assumption was confirmed after checking for nonzero slope of scaled Schoenfeld residuals on time ^41^.

**References Supplementary**

^38-41^

38. de Biase D, Acquaviva G, Visani M *et al.*Molecular diagnostic of solid tumor using a next generation sequencing custom-designed multi-gene panel. *Diagnostics (Basel)* 2020;10.

39. Kopanos C, Tsiolkas V, Kouris A *et al.*Varsome: The human genomic variant search engine. *Bioinformatics* 2019;35;1978-1980.

40. Leon-Castillo A, Britton H, McConechy MK *et al.* Interpretation of somatic POLE mutations in endometrial carcinoma. *J Pathol* 2020;250;323-335.

41. Grambsh PM, Therneau TM. Proportional hazards tests and diagnostics based on weighted residuals. *Biometrika* 1994;81;515–526.

**Supplementary Figures**

**Supplementary Figure 1.** Kaplan–Meier estimates of disease-free survival according to HLA class I expression in the entire cohort; censoring times are marked with red spikes.


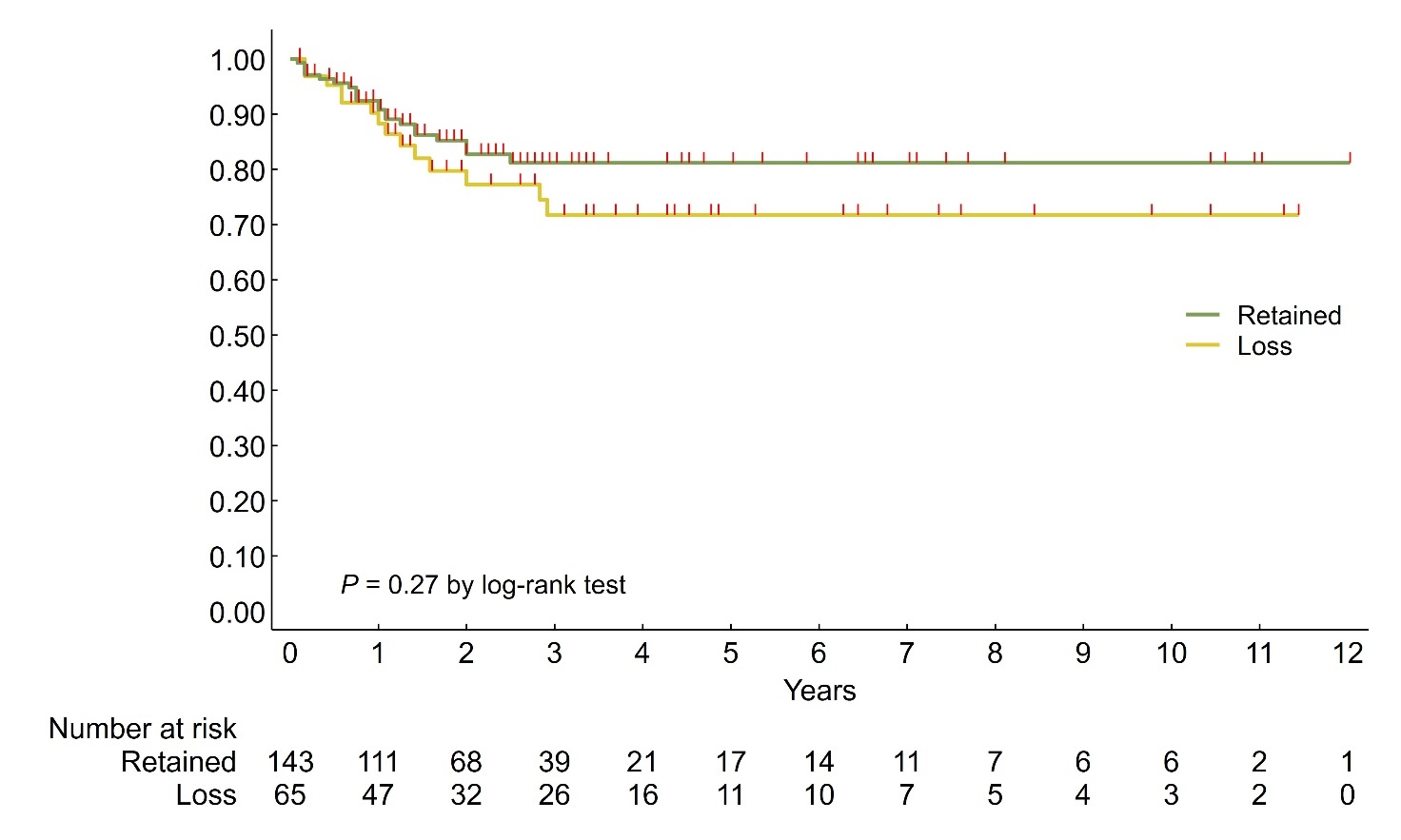


**Supplementary Figure 2.** Kaplan–Meier estimates of disease-free survival restricted to FIGO2009 stages I and II (*n* = 161) according to HLA class I expression; censoring times are marked with red spikes.


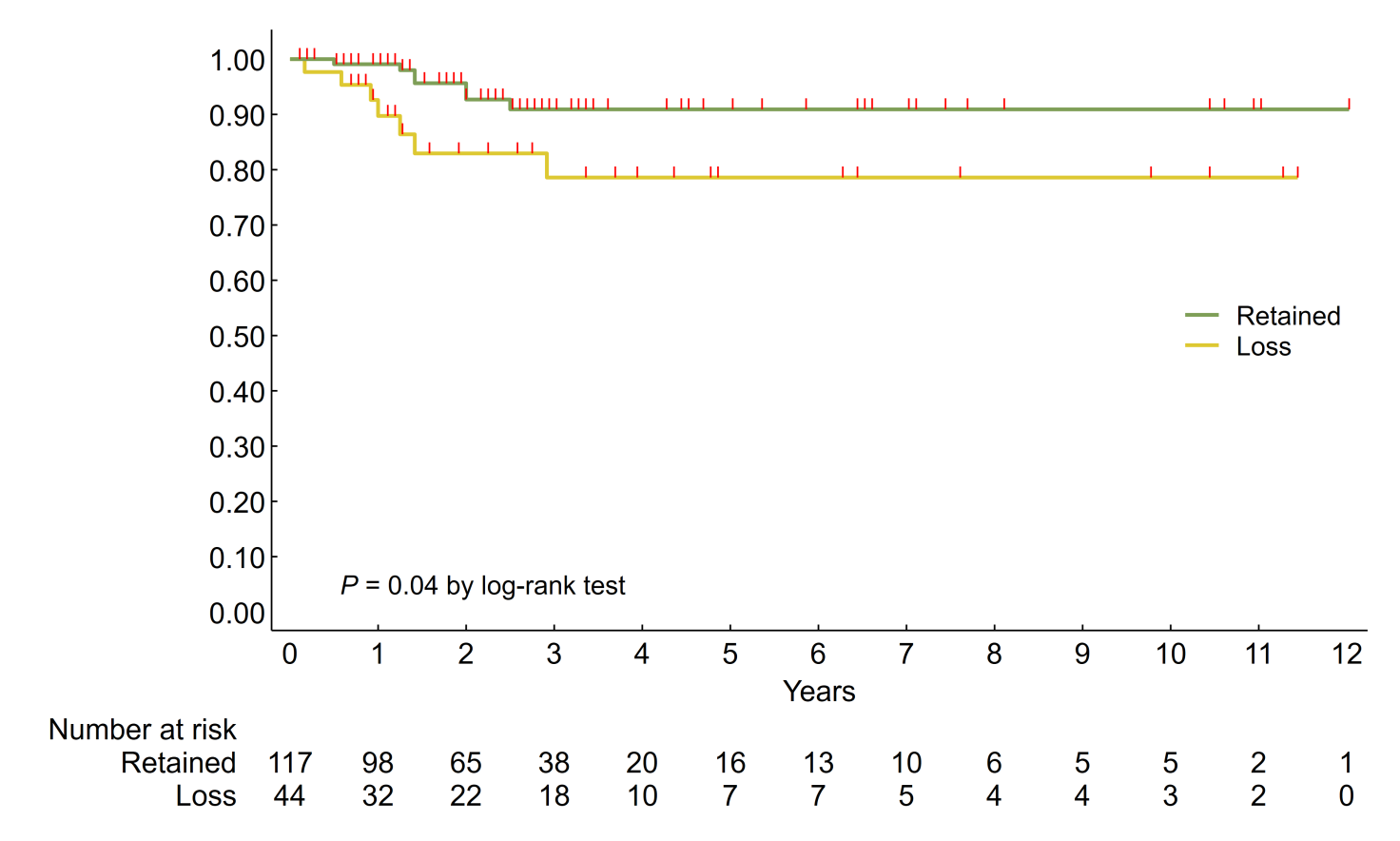

Supplement: Supplementary file 1 — Figure S1. Kaplan–Meier estimates of disease‐free survival according to HLA class I expression in the entire cohort; censoring times are marked with red spikes. Figure S2. Kaplan–Meier estimates of disease‐free survival restricted to FIGO 2009 stages I and II (n = 161) according to HLA class I expression; censoring times are marked with red spikes. Table S1. List of antibodies and protocols. Supplementary Material and Methods. [file HIS-88-442-s001.docx]
